# Sonographic Findings in Partial Type of Trisomy 18

**Published:** 2013-12-22

**Authors:** Maryam Niknejadi, Firoozeh Ahmadi, Farnaz Akhbari, Parvaneh Afsharian

**Affiliations:** 1 Department of Reproductive Imaging at Reproductive Biomedicine Research Center, Royan Institute for Reproductive Biomedicine, ACECR, Tehran, Iran; 2 Department of Genetics at Reproductive Biomedicine Research Center, Royan Institute for Reproductive Biomedicine, ACECR, Tehran, Iran

**Keywords:** Trisomy 18, Fetal Ultrasonography, Congenital Abnormality

## Abstract

Trisomy 18 (Edwards syndrome) is the second most common trisomy among live born fetuses, with poor prognosis. Estimate of its incidence is between 1 in 4000- 16000 live births. Most of the chromosomal abnormalities in fetuses are detected by prenatal ultrasound findings in the first and second trimesters. In this case re- port, we present a partial type of trisomy 18 occurring through de novo unbalanced translocation of chromosomes 18 and 21. The ultrasound features enabling the early detection of trisomy 18 include a delayed ossification of calvarium combined with early onset of fetal growth restriction (FGR) and the absence of nasal bone through performing triple test followed by amniocentesis. Finally, the parents decided to terminate the pregnancy.

## Introduction

After Down syndrome, Edward syndrome (Fig trisomy 18) is the second most common trisomy among live born fetuses (1, 2). Clinicians strive to detect any prenatal chromosomal abnormalities early in the pregnancy. This is especially true for conditions involving lethal trisomies, such as trisomy18, in which prenatal care may totally change after diagnosis. It is a fatal disease with very poor prognosis. Ninety percent of affected new born die during the first year of life and the remaining 10% suffer from severe mental retardation. Edwards syndrome is associated with profound neurological damages and mental deficiency in these neonates, who have an average life expectancy of one week (3, 4). The only definitive methods to make a diagnosis of trisomy 18 are through ultrasound imaging, particularly during the first and second trimesters, triple tests and invasive testing with amniocentesis or chorionic villous sampling (1, 5, 6). Amniocentesis has been associated with an increased risk of miscarriage; therefore, it is usually offered for high risk patients.

For the detection of trisomy 18, ultrasound findings in the first and second trimester for trisomy 18 seems to be more effective than biochemical screening. In order to achieve a more accurate diagnosis of trisomy 18, we must combine sonography, triple test and amniocentesis (1, 3, 5). Sensitivity of ultrasound screening for trisomy 18 was reported 70% (7), while a multiple marker test [Alpha fetoprotein, human chorionic gonadotropin (HCG), Unconjucated estriol] was abnormal only in 43% of cases with trisomy 18 (8).

## Case Report

A 26 year-old woman, gravid 1, para 0, abortion 1 and two years primary infertility was admitted to our infertility clinic at the Royan institute, Tehran, Iran. She had an eight-week missed abortion followed by dilatation and curettage (D&C) in 2006. Physical history included severe polycystic ovarian syndrome (PCO), hypothyroidism, diabetes mellitus and obesity. The couple’s karyotypes were normal. Semen analysis obtained from the husband showed low volume (0.2 cc), low motility (8%) and abnormal morphology (92%); in addition, he suffered from severe oligospermia. After intracytoplasmic sperm injection (ICSI), a normal live fetus was revealed by initial transvaginal sonography at 7.5 weeks. At 12.5 weeks of gestation, ultrasonography determined normal nuchal translucency (NT) measurement with early onset of fetal growth restriction (FGR) which was compatible with the result obtained at 11 weeks of gestation according to crownrump- length (CRL); in addition, the nasal bone and some part of calvarium were absent (Figes1, 2). At 14 weeks, the parietal part of calvarium was formed and nasal bone was seen partially. At this time, FGR became more severe, and differences among biometric measurements [biparietal diameter (BPD)], femor length (FL) and real gestational age had increased (Fig 3). Therefore, a triple test was carried out on the parents, which was followed by amniocentesis. The result of the triple test indicated a high risk of trisomy 18 (1/99), which was confirmed by amniocentesis. The karyotype 46 xy der (20) t (18:21, q10: q10) was found in cultured amniotic cells which was compatible to a male fetus with trisomy of long arm of chromosome 18. At that time, the parents decided to terminate the pregnancy at 18 weeks of gestation.

**Fig 1:**
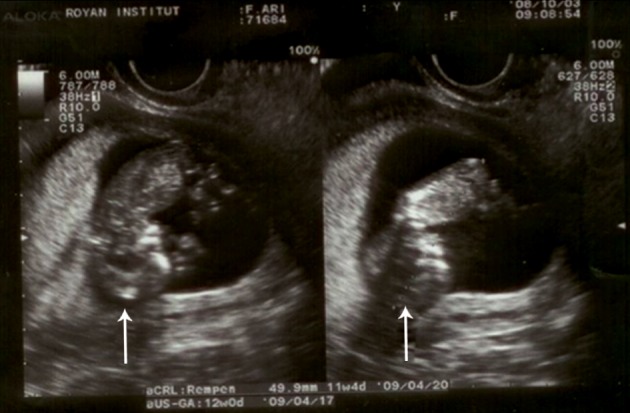
First trimester transvaginal ultrasonography in a 26- year old pregnant woman at 12.5 weeks of pregnancy which is compatible with results obtained at 11-11.5 weeks of pregnancy based on CRL measurement. Most part of calvarium bone cannot be observed, and nasal bone is not detectable, as well.

**Fig 2:**
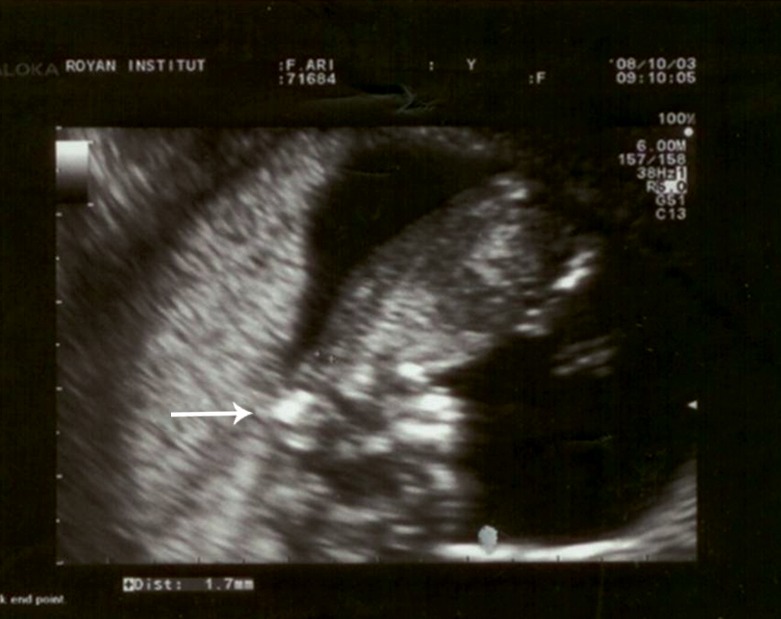
First trimester sonography in a 26-year old pregnant woman at 12.5 weeks of gestation. The undetectable calvarial bone can be observed clearly in this image, which confirms previous diagnosis.

**Fig 3:**
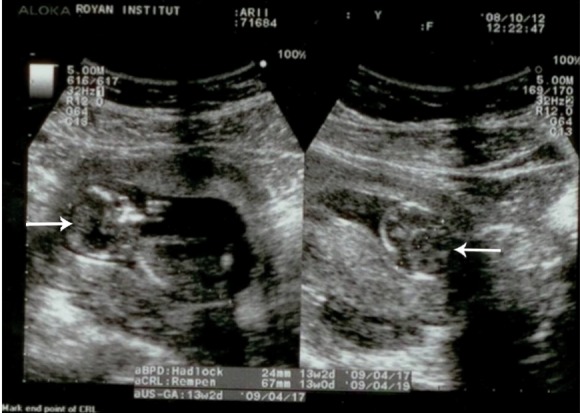
This is a transabdominal sonography in a 26-year old pregnant women. As shown, fetal growth restriction and partial calvarium bone can be detected at 14 weeks of pregnancy. Biometric measurement is compatible with the findings at 13-13.5 weeks of pregnancy.

## Discussion

There are two types of trisomy18 including partial and complete, whereas in 80% of cases, there is complete trisomy, and in 20% of cases, a partial trisomy can only be detected, as a consequence of various abnormalities of chromosome 18 such as duplication, additional isochoromosomes of short or long arm of chromosome 18, as well as translocations involving chromosome 18 and other autosomal chromosomes can be resulted in partial trisomy 18 (9, 10). Since in this case, the both parents’ karyotypes were normal, we assumed this partial trisomy 18q is due to a de novo unbalanced translocation of chromosomes 18 and 21, of which only a few cases have been reported.

Approximately, three-quarters of pregnancies with the fetus diagnosed with trisomy 18 result in a miscarriage or stillbirth between the 12th week of gestation and term (11). Postnatally, the median survival time is 3-6 days. Less than 50% of infants will survive for a week, and only, about 5-10% will survive for a year (12, 13). Long term survival in trisomy 18 has been reported, but this occurs primarily in the context of mosaicism (14, 15).

Recently, Koide et al. (16) have published the in utero gene expression profile of second trimester fetuses affected with trisomy 18. According to the results, 251 genes showed significant differential expression in cases with trisomy 18 compared to the controls, but only 7 genes out of 251 were located on chromosome 18.

Fetal sonographic findings which have been relevant to trisomy 18 include congenital heart disease, mainly ventricular septal defect (VSD, 17), choroid plexus cysts, gastrointestinal disease such as diaphragmatic hernia and imperforated anus, microcephaly, microphthalmia, omphalocele, kidney abnormalities, early-onset of fetal growth restriction (FGR) and pyelectasis 4 mm (17, 18). Reported skeletal dysmorphologic signs include limb abnormalities, polydactyl, absent fibula, radial aplasia, clenched hands, and rocker bottom feet which mostly are seen in partial types. The findings of prospective studies have demonstrated that there is an absence of the nasal bone in almost 50% of the fetuses with trisomy18 at 11-13 weeks of pregnancy (18-20). In this case, we observed a delayed ossification of calvarium and the absence of nasal bone together with partial trisomy 18q. Such a case has not been reported before, so we present this subject for the first time within partial trisomy 18q. Despite all of the skeletal sonographic findings, in this case, the two mentioned features were only detected.

Most fetuses with trisomy 18 have an abnormal ultrasound results. This doesn’t mean that all anomalies can be identified through an ultrasound, but at least, one anomaly can be seen in the majority of cases (1).

With the improvement in availability of first trimester ultrasound, enhanced resolution image and better sonographic techniques, many of the anomalies related to trisomy 18 can be systematically detected at the early stages of pregnancy.

More studies need to be done in order to know whether calvarium bone screening in early sonography can be an important characteristic for the diagnosis of trisomy 18.
